# Comparison of Spa Choice between Wellness Tourists and Healthcare/Medical Tourists

**DOI:** 10.3390/healthcare8040544

**Published:** 2020-12-09

**Authors:** Jorge Pelegrín-Borondo, Noelia Araújo-Vila, Jose A. Fraiz-Brea

**Affiliations:** 1 Economics and Business Department, University of La Rioja, 26006 Logroño, Spain; jorge.pelegrin@unirioja.es; 2 Faculty of Business and Tourism, University of Vigo, 32004 Ourense, Spain; jafraiz@uvigo.es

**Keywords:** wellness, spas, healthcare tourists, decision making

## Abstract

Spa tourism is considered one of the most important segments of the $639 billion wellness market. The literature refers to two types of spa tourists: wellness tourists and healthcare/medical tourists. However, virtually no studies have compared spa choice models between these two segments. The present study uses the Cognitive-Affective-Normative (CAN) model to compare the variables that explain purchase intention in relation to spas between the two segments. Data were collected through a questionnaire administered to a sample of 810 potential Spanish spa-goers, and consistent partial least squares (PLSc) structural equation modeling (SEM) was used. Contrary to what might be expected, no major differences were found between the spa choice models for wellness tourists and for healthcare/medical tourists. The results show that R2 and Q2 were similar for both models. The most influential variable was performance expectancy, and differences were only found in the influence of the pleasure variable.

## 1. Introduction

According to the Global Wellness Institute [1], wellness tourism is a $639 billion market. Although the same Institute originally expected it to reach $919 billion by 2022, due to the Covid-19 pandemic, it is no longer on track to meet that forecast. According to the World Tourism Organization (UNWTO) [2], tourism will not return to its pre-Covid-19 levels for another 2 or 3 years. Nevertheless, in 2017, the wellness tourism market grew 5.4%, almost twice as fast as general tourism (3.2%). That year, it accounted for 830 million trips and 17% of total tourism expenditures [1]. Furthermore, international wellness tourists spend an average of $1528 per trip, 53% more than the typical international tourist; likewise, domestic wellness tourists spend $609 per trip, 178% more than the average domestic tourist [3].

The wellness industry, which includes spas, is a growing multi-trillion-dollar-a-year global industry [4]. In this context, the spa (or thermal spring) market is particularly promising, as one of the largest business segments of the wellness tourism industry [5,6]. This spa market includes significant demand for relaxation [7,8] and stress alleviation [9,10,11,12]. However, the market’s growth is also driven by tourists’ increasing interest in the health and wellness benefits of water-based therapies [6]. The literature thus recognizes the existence of two main segments of spa tourists based on the core benefit sought: wellness spa tourists and healthcare/medical spa tourists [13,14]. Spas meet the requirements of both medical and wellness tourists. The former require treatments for specific medical reasons while the latter seek to preserve their health [15]. Knowing how these segments behave when it comes to choosing a spa can be crucial to enabling businesses that provide these services to develop appropriate strategies.

Despite the importance of spa tourism and the existence of these two large established segments based on the benefits sought, few studies have looked at wellness tourists and healthcare/medical tourists together [14,16], and, to the authors’ knowledge, virtually none has compared their spa choice models. The present research aims to help fill this gap by comparing how the variables of the Cognitive-Affective-Normative (CAN) model [17] influence wellness tourists and healthcare/medical tourists to explain spa choice. Specifically, it aims to answer the question: Do the explanatory variables affecting the intention to purchase a spa service influence wellness tourists and healthcare/medical tourists (two segments seeking different benefits) the same way?

The CAN model is an extension of the Unified Theory of Acceptance and Use of Technology (UTAUT) and Technology Acceptance Models (TAM). The Unified Theory of Acceptance and Use of Technology (UTAUT) model [18] and its extension, the UTAUT2 model [19], are general models explaining technology acceptance. They are essentially based on the Technology Acceptance Models (TAM and TAM2) [20,21,22], which, in turn, are based on the Theory of Reasoned Action (TRA) [22] and the Theory of Planned Behavior (TPB) [23]. Both the UTAUT models and their predecessors, the TAM models, have been widely used in the study of technology acceptance.

The CAN model contains far fewer observable variables than the UTAUT model and is more suited to products in which the affective component is important. This is because the CAN model includes affective-emotional variables in the modeling, which, together with cognitive and normative factors taken from the earlier models (TAM and UTAUT), help explain users’ intention to use a product. This model has been applied to both technological [24] and non-technological [25,26,27] products, with good results, Pelegrín, J. ts in terms of its explanatory power.

For the present research, emotional variables from the CAN model were modified. The basic emotions used in the original model by Pelegrín-Borondo et al. [17] were replaced with the emotional dimensions of pleasure and arousal established by Mehrabian and Russell [28] and Russell and Mehrabian [29] for the reasons explained in the sub-section “Influence of emotions on spa purchase intention” below.

## 2. Hypothesis Development

### 2.1. The Influence of Performance Expectancy and Effort Expectancy on Spa Purchase Intention

Performance expectancy is defined as the degree to which a person considers that using a specific technology would be useful to enhance what matters to that person, while effort expectancy is the degree of ease associated with the use of a specific technology [18]. Behavioral intention is defined as the degree to which a person plans to perform or not perform a specific behavior [22]. In the present research, tourists’ spa purchase intention refers to the degree to which a person plans to go or not go to a spa.

Usefulness has been recognized as an important variable in the decision to go to a spa. Valentine [30] highlights the usefulness of natural remedies based on air, water, rest, and healthy diets as a key factor for visiting a spa. Similarly, other authors have found that customers decide to go to spas because they consider them useful for improving their health [31,32,33]. Some authors have also found that spas are considered a useful place for socializing and building relationships [7,31].

Although the literature has recognized the positive influence of ease of use on technology acceptance by tourists [34], few studies have examined this variable in relation to spas. Alén, Fraiz, and Rufín [35] highlight “friendly treatment” as one dimension of Spanish spa-goers’ expectations. Chiu and Ku [36] find an association between expected effort and greater use of healthcare technologies.

In light of these earlier findings, the following hypotheses are proposed:

**Hypotheses 1 (H1).**  *Performance expectancy regarding a thermal suite positively affects potential spa tourists’ purchase intention.*

**Hypotheses 2 (H2).**  *Effort expectancy regarding a thermal suite positively affects potential spa tourists’ purchase intention.*

### 2.2. Influence of Emotions on Spa Purchase Intention

Under Scherer’s [37] componential theory of emotion, emotion is defined by the joint existence of a series of traits, namely: a stimulus triggering the emotion; the possibility of attributing that stimulus to a specific cause; the generation of a characteristic physiological response; the existence of a cognitive assessment (as opposed to a visceral response); feelings of pleasure-displeasure; a qualitatively unique feeling; a tendency toward a characteristic action; and the short-lived nature of the process [38]. Emotions are widely recognized to push consumers to act [39,40]. Sharma and Nayak [41] showed that that tourists’ emotions positively influenced overall image and satisfaction and both overall image and satisfaction positively influenced behavioral intentions in wellness tourism.

In general, the literature has established two main approaches to the analysis of emotions: (i) the categorical approach, which analyzes the basic emotions a person is feeling, such as anger, sadness, fear or happiness; and (ii) the dimensional approach, which seeks a set of emotional dimensions, the intersection of which makes it possible to determine how a person feels.

Some authors have advised against using the categorical or basic emotion approach, as it is premised on the notion that emotions are easily recognizable for people and well defined in an individual’s mind and that people can easily differentiate between them [42,43,44]. In fact, people have trouble establishing the limits to distinguish between certain emotions [45,46,47]. Additionally, it is not easy to define a limited number of basic emotions that includes all possible options [48], since emotions are like colors: they are infinite in number and infinitely nuanced.

While the categorical approach represents each of the emotions a consumer may feel, the dimensional approach represents reflections or consequences of the emotion an individual is feeling, which are included in dimensions that make it possible to analyze that emotion. In this regard, Mehrabian and Russell [28] and Russell and Mehrabian [29] show that it is possible to establish what a person is feeling using a limited number of emotional dimensions. These authors propose a scale with three dimensions: pleasure, arousal, and dominance (PAD). Eroglu et al. [49] note that “in many instances, the dominance dimension is not included, probably due to Russell’s [45] recommendation that pleasure and arousal alone can adequately represent the range of emotion exhibited in response to environmental stimuli.” Accordingly, there is a certain consensus surrounding the use of the arousal and pleasure dimensions to dimensionally measure an individual’s response to a stimulus [38].

The tourism literature reflects the importance of both pleasure and arousal in the study of tourist behavior [24], determining that both dimensions positively influence tourist behavior [50,51,52].

Specifically, in relation to spas, pleasure is considered one of the most important motivating factors for going to them. Hsieh [31] concludes that feeling good is one of the four main motivations for Taiwanese spa tourists. Huh, Lee, and Lee [53] identify a spa-goer market segment that they call “pleasure pursuers,” which is the largest segment in their study. Kucukusta and Denizci Guillet [32] also identify a segment of pleasure-oriented spa-goers. Han, Thuong, Kiatkawsin, Ryu, Kim, and Kim [54] use the emotional dimensions of pleasure and arousal in their study on customers’ intention to return to a spa hotel. They show that pleasure significantly and positively influences behavioral intention but find no similar influence for arousal.

Based on these findings, the following hypotheses are proposed:

**Hypotheses 3 (H3).**  *The pleasure produced by the idea of going to a spa positively affects potential spa tourists’ purchase intention.*

**Hypotheses 4 (H4).**  *The arousal produced by the idea of going to a spa positively affects potential spa tourists’ purchase intention.*

### 2.3. Social Influence on Spa Purchase Intention

In the context of new technologies, social influence is defined as the degree to which an individual perceives that important others believe that he or she should use a given technology [18]. This perception represents the social pressure to engage or not engage in a given behavior [24]. Social influence has also been shown to be important in tourist behavior [55,56].

Specifically, in the context of spas, the opinions of others have been shown to play a decisive role in the choice to go to a spa. Klaysung [57] finds that positive reviews from friends are important in spa choice. Similarly, Kamenidou et al. [33] identify recommendations from friends or family as an essential motivating factor for going to a spa. Ordabayeva and Yessimzhanova [14] show that friends’ recommendations are the main reason for visiting a sanatorium-resort institution (including spas). In contrast, Kim, Kim, Huh, and Knutson [58] do not find social norms to have a significant influence on the intention to visit a spa.

In light of these earlier findings, the following hypothesis is proposed:

**Hypotheses 5 (H5).**  *Social influence in favor of a thermal suite positively affects potential spa tourists’ purchase intention.*

### 2.4. Moderating Influence of the Core Benefit Sought: Wellness Spa Tourism vs. Healthcare/Medical Spa Tourism

The core benefits sought are the main reason or reasons for consuming a given product [59]. The literature has long recognized the powerful potential of segmentation by sought benefits in the tourism product market [60].

Carrera and Bridges [61] define “health tourism” as organized travel outside one’s local environment for the purpose of maintaining, enhancing, or restoring one’s wellbeing in mind and body. The health tourism segment can be further divided into two subtypes based on the core benefits sought: wellness tourism and healthcare/medical tourism [15,62,63]. Alegría Quintela, Costa, and Correia [16] identify two groups of sought benefits that can be observed in health tourism from a conceptual point of view: (i) therapeutic benefits, associated with healthcare/medical tourism, which include therapeutic treatment to cure and/or prevent diseases; and (ii) recreational benefits, associated with well-being or wellness tourism, focused on relaxation, leisure, and escape from routine. Similarly, Ordabayeva, and Yessimzhanova [14] note that “healthcare and wellness tourism can be divided into therapeutic tourism, aimed at treatment, therapy and rehabilitation after diseases, and wellness-tourism, aimed at maintaining [the] human organism fit, as well as maintaining balance between [the] physical and psychological health of a person. Wellness-tourism, in its turn, can be active (sport and fitness) and passive (beauty programs).” The identified motives for going to a spa can also change. Trips are not always made for healing purposes, but sometimes for prophylactic or even recreational ones [64,65].

In the context of the spa market in particular, in the mid-1800s, the Spanish physician Nicolás Escolar (1865) complained about people who feign illness to enjoy a spa. In this regard, Escolar was already distinguishing between two major benefits sought from a spa: wellness/enjoyment and healthcare/medical benefits. In a study on spa tourism in Spain, Vázquez-Illá [13] finds that the two benefits most often cited by spa-goers are: (i) to alleviate stress and relax (48.9%); and (ii) to alleviate pain and cure diseases (15.3%). Likewise, Costa et al. [66] highlight the following among the main reasons for visiting a spa region in Portugal: health (40.1%) and wellness/wellbeing (27.9%). The importance of these two core benefits sought at spas and of their associated segments (wellness and healthcare/medical) [6,53] has been highlighted in numerous studies in several countries, including Finland [67], Greece [33], Jamaica [30], Japan [68], Taiwan [31,69], and the U.S. [7].

The spa benefits related to wellness generally include relaxation, socializing, beauty, and escape from routine [33,70,71,72,73]. In this regard, the type of wellness associated with tourism has been defined as “a phenomenon to enhance personal well-being for those traveling to destinations which deliver services and experiences to rejuvenate the body, mind, and spirit” [74]. Wellness is often associated with tasteful, trendy, and stylish products in the media [73]. With regard to the healthcare/medical benefits obtained at spas, Koh et al. [7] conclude that the health benefits and rejuvenation are the most important spa-selection criteria for spa tourists in the U.S. Hsieh [31], Kucukusta and Guillet [32], and Kamenidou et al. [33] highlight health improvements through spa treatments as one of the essential reasons for going to spas, while Kamenidou et al. [33] identify therapeutic reasons and body care as motivational factors for spa tourists.

Notwithstanding this literature, few studies compare spa choice between these two segments. Boekstein [75] establishes that the international thermal-spring health tourism product has undergone significant changes, including declining demand for the medically-oriented services offered by traditional spas and increased demand for facilities, services, and experiences geared toward wellness, often accompanied by a greater focus on recreation.

**Figure 1:**
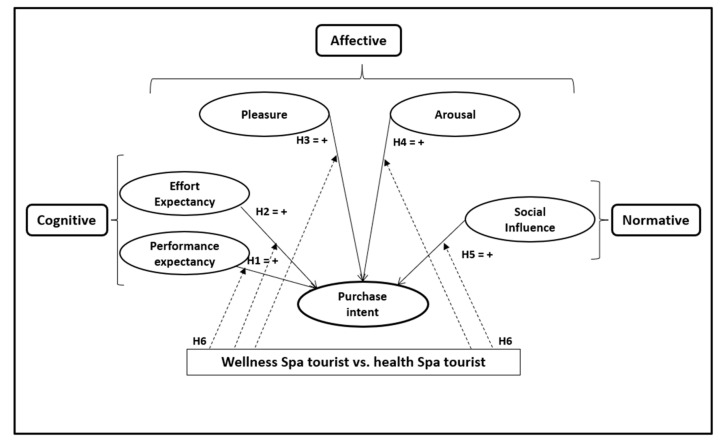
Proposed hypothesis model.

Given the scant literature comparing the wellness and healthcare/medical spa tourism segments, and in light of the aforementioned studies, the following proposition is made (Figure 1).

**Hypotheses 6 (H6).**  *The core benefit sought–wellness vs. healthcare/medical–moderates the influence of the explanatory variables affecting potential spa tourists’ purchase intention.*

## 3. Methodology

### 3.1. Design

The Oca Augas Santas Balneario and Golf Resort spa (Pantón, Lugo, Galicia) was selected as the spa for collecting the study data. It was chosen for its location, as Galicia is home to a large concentration of spas; along with Catalonia, it is the Spanish region with the largest number of such establishments (21). It also has numerous hot springs (more than 300) and is the top Spanish spa destination [76]. Additionally, the spa itself is located in A Ribeira Sacra, a leading destination in Galicia due to its landscape, heritage, and wine tourism, which has applied for World Heritage status. Moreover, Spain is one of 12 European countries included in the 2017 ranking of the world’s top 20 wellness tourism destination markets, ranking 15th, with 18.8 million trips. Likewise, Europe receives more wellness tourism trips than any other continent in the world, registering a total of 292 million trips in 2017 [1].

Because the structure of the Spanish spa-going population for any reason (wellness tourism or healthcare/medical tourism) is unknown, it was decided to look for an evenly distributed sample in terms of gender and age (Table 1). Other researchers have followed similar strategies [25,77]. Specifically, a sample of potential tourists was sought stratified by gender (with an equal number of men and women) and age (distributed equally across five age brackets). The data were collected according to the following procedure. Trained interviewers contacted people from their own personal networks who fit the predefined gender and age profiles and were not residents of the spa’s location. A snowball-sampling technique was then used, whereby the people first contacted were then asked to provide the contact details of other people living anywhere in Spain who also fit the required profile to take the survey. The interviewers conducted the surveys in person or by phone, after arranging an interview. Prior to administering the survey, the interviewer showed each respondent a 45-s video featuring images of the hotel (exterior locations, rooms, golf course), the thermal suite (outdoor and indoor spa facilities), and the price of using the thermal suite. When the survey was administered by phone, the video and offer were sent in advance by e-mail to the respondent’s computer or to his or her phone. In this regard, a greater effort was required for the oldest age group. At the start of the interview, the interviewer said, “Think about the thermal circuit you have seen in the video. Imagine that you were going to purchase a trip there in the future. What would be your main motivation for going?”. The interviewee had to choose between wellness or healthcare/medical reasons. Subsequently, the interviewer asked the survey questions and thanked the respondent for his or her participation. Based on this sample, those surveys presenting inconsistencies or errors were discarded, resulting in a final total sample of 810 people, the characteristics of which are shown in Table 1.

### 3.2. Measures

Table 2 shows the constructs, the scales used, and the bibliographic sources from which the scales were taken. The scales for the variables performance expectancy, effort expectancy, and social influence were adapted from the UTAUT2 model [21]. The scale for purchase intention was adapted from the TAM model [21]. The observable variables were measured on Likert scales ranging from 0 to 10.

The original measurement scale for the emotional dimensions of arousal and pleasure [28,29] has been modified numerous times in the literature to ensure a better fit for research. Miniero, Codini, Bonera, Corvi and Bertoli [78] consider that the PAD scale might be successfully reduced, gaining scale reliability. Based on these considerations, the present research uses the selection of PAD scale items made by Loureiro [79] in application to the tourism industry. In this case, the survey respondents were asked to consider how they felt when thinking about the thermal suite and to rate each adjective from the emotional dimensions accordingly on a semantic differential scale, ranging from −5 to 5.

### 3.3. Statistical Analysis

The collected data were analyzed using structural equation modeling (SEM). More specifically, the consistent partial least squares (PLSc) SEM technique was used. Unlike partial least squares (PLS), PLSc is less sensitive to Type I and Type II errors and should be applied to models in which all the constructs are reflective [80], as in the present case. Additionally, PLS tends to skew factor loadings upwards and underestimate regression coefficients [81]. Partial least squares SEM techniques (both PLS and PLSc) are less sensitive to the violation of assumptions of data normality than other SEM techniques [82]. Furthermore, PLSc is appropriate for research seeking to predict or explain a phenomenon [83], as is the case here.

To test the proposed hypotheses, a sequential statistical process was followed:

- Step 1: Assessment of the measurement models. Two models were established: the first includes the influence of the CAN model variables on wellness tourists’ spa purchase intention; the second includes the influence of the CAN model variables on healthcare/medical tourists’ spa purchase intention. For the database for each model, the measurement model was assessed by testing the reliability and validity of the measurement scales. Given that for multigroup tests to be comparable, they must use the same model (configural invariance criterion), when an observable variable had to be eliminated, it was eliminated from both models.
- Step 2: Assessment of the structural model. For each of the two models, the R2, path coefficients, and their significance were estimated. For each model, the influence of the CAN model variables on spa purchase intention was tested.
- Step 3: Multigroup comparison of the models. The structure of the two models (wellness and healthcare/medical) was identical, making it possible to proceed to a multigroup comparison. The factorial invariance between the measurement models was verified [82]. For the multigroup comparison, the non-parametric PLS-MGA test proposed by Henseler et al. [82] was performed to determine whether the influence of the different CAN model dimensions on wellness and healthcare/medical tourists’ spa purchase intentions was different or similar.

## 4. Results

### 4.1. Assessment of the Measurement Models

First, the reliability of the wellness and healthcare/medical model indicator was analyzed. The PLSc SEM technique results indicated that the standardized loadings of the observable variable AR2 (Calm–Excited) in the healthcare/medical model interfered with the indicator’s reliability, so this variable was eliminated from both models. The variable EE4 had loading values of <0.7, but its *t*-value was >1.96. The 0.7 standardized loading rule is flexible, particularly when indicators contribute to a factor’s content validity, so this item was kept in both models. As all standardized loadings were higher than 0.7 and all *t*-values higher than 1.96 for both of the resulting models (wellness and healthcare/medical), the indicator’s reliability was good (see Table 3).

Table 4 shows that the reliability was good for both models, with a Cronbach’s alpha and composite reliability >7. The convergent validity criterion was also met, as the average variance extracted (AVE) for both models was greater than 0.5. Both models likewise met the discriminant validity criterion: the HTMT values were correct in all cases (<0.9), and the square root of the AVE was greater than the correlations among constructs.

### 4.2. Assessment of the Structural Model

**Figure 2:**
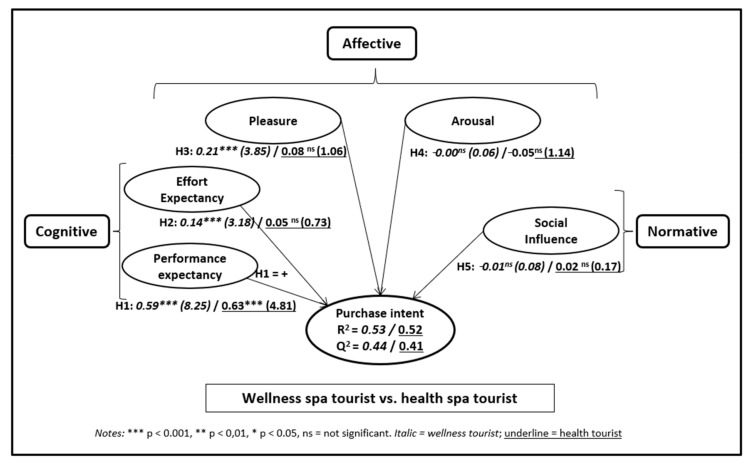
Wellness spa vs. health spa tourist results.

Figure 2 shows the R2 and Q2 obtained with PLSpredict, the path coefficients (direct effect), and the Student’s t-test and p-value for each antecedent variable of spa purchase intention, showing the results for both wellness tourists (italics) and healthcare/medical tourists (underlined).

R2 was 0.53 for the purchase intention model for wellness tourists and 0.52 for the model for healthcare tourists. In both models, Q2 was greater than 0; specifically, it was 0.44 in the wellness tourist model and 0.41 in the healthcare/medical tourist model. This indicates that the exogenous variables do indeed relevantly predict the endogenous variable and that the predictive power of both models is similar. 

In both the wellness tourist model and the healthcare/medical tourist model, the variable performance expectancy significantly influenced spa purchase intention; in both cases it was the variable with the greatest impact on purchase intention. Support was thus found for Hypothesis H1.

The effort expectancy and pleasure variables had a significant influence on purchase intention for wellness tourists but not healthcare/medical tourists. Therefore, only partial support was found for Hypotheses H2 and H3.

Neither arousal nor social influence affected spa purchase intention. Therefore, no support was found for Hypotheses H4 and H5.

### 4.3. Multigroup Analysis

In order to perform a multigroup analysis to examine the differences in the influence of the CAN model dimensions on purchase intention between the wellness and healthcare/medical tourist models, the measurement invariance was first tested: (i) the two models have the same structure, so the configural invariance condition was met; (ii) among the standardized values of the observable variables, obtained from the PLSc analysis, the significance of Levene’s test of equality of variances was greater than or equal to 0.1 (the lowest value was 0.1), indicating that there are no statistically significant differences; and (iii) among the standardized values of the observable variables, the significance of the independent samples t-test was >0.1 in all cases (the lowest value was 0.4), meaning there are no statistically significant differences between the means of the standardized variables. There were thus no problems in terms of measurement invariance.

The non-parametric PLS-MGA test proposed by Henseler et al. [82] was used for the multigroup analysis, using the bootstrapping results obtained from the PLSc analysis. Table 5 shows the results of this non-parametric test. The test shows that the only statistically significant difference between the wellness and healthcare/medical models is in the influence of the variable pleasure on spa purchase intention. Other than the pleasure variable (for which only a moderate level of difference was detected between the two models), no large differences were found to exist between wellness tourists and healthcare/medical tourists in terms of the influence of the explanatory variables.

## 5. Discussion

This research compares spa choice between wellness tourists and healthcare/medical tourists, applying the Cognitive-Affective-Normative (CAN) model [17]. The application of this model shows a good fit and predictive power in both cases (wellness spa tourists and healthcare/medical spa tourists). R2 was 0.53 for wellness tourists and 0.52 for healthcare/medical tourists. Q2 was 0.44 for wellness tourists and 0.41 for healthcare/medical tourists. These similarities in the explanatory power are the first result indicating that the two tourist segments are equal. As will be discussed below, other results also point to this equal nature.

Surprisingly, no significant differences were found between the two models (wellness tourists and healthcare/medical tourists) in the influence of the explanatory variables of spa purchase intention, except in the variable pleasure. For both wellness spa tourists and healthcare/medical spa tourists, the variable with the strongest influence on purchase intention is performance expectancy. This finding is consistent with prior studies that have shown the importance of this variable for spa tourists [30,31,32,33], but it qualifies those earlier findings insofar as it shows that performance expectancy is the most important variable for both wellness spa tourists and healthcare/medical spa tourists. It should be noted that, for the healthcare/medical spa tourist segment, performance expectancy was the only variable to have a significant effect on purchase intention. This seems logical since, when a person is sick, the need to get better is virtually the sole reason to go to a spa.

## 6. Conclusions and Implications

The second most influential variable with regard to spa purchase intention in wellness tourists was the emotional dimension pleasure. This variable was not significant for healthcare/medical spa tourists. The multigroup test shows that there are significant differences in the influence of pleasure between the two segments. This finding is logical, since wellness is associated with pleasure-seeking and enjoyment [53,73], and a spa is an ideal place for that. In contrast, healthcare/medical tourists seek to improve their medical problems, and pleasure is not one of their main motivations. The prior findings of Lee [69] and Kucukusta et al. [33], who identified a market segment of pleasure-seeking spa-goers, were on the right track. However, while pleasure is important for wellness spa tourists when it comes to choosing to go to a spa, the most important aspect for them is its usefulness.

The influence of the variable effort expectancy was significant for wellness spa tourists but not for healthcare/medical spa tourists; however, the difference in this parameter between the two groups was quite minor, so the multigroup test did not show significant differences. Prior work on the influence of this variable in the context of spas is scarce. Unfortunately, the present results do not shed much light on the issue, as effort expectancy was only the third most influential variable in the wellness tourist segment and had no influence on the healthcare/medical tourist segment.

Neither the emotional dimension arousal nor social influence were observed to have a significant influence in either segment. In this regard, earlier findings establishing the importance of arousal in tourism [21,24,50] did not hold in the context of spa tourism. The present findings corroborate the work of Han et al. [54], who found that arousal did not have any influence on customers’ intention to return to a spa hotel. Nor was it possible to corroborate studies establishing the influence of friends and family members [33,57] on the intention to go to a spa.

The most obvious recommendation for spa managers is that, if they wish to attract customers from both segments (i.e., wellness tourists and healthcare/medical tourists), they should focus on conveying the usefulness of going to their spa. However, as noted, the literature shows that the content of this usefulness can differ. For wellness tourists, it refers to relaxation, socializing, beauty, stress alleviation, and escape from routine, while for healthcare/medical tourists, it refers to pain alleviation, treating diseases, and improving health. The marketing for each segment should thus focus on these uses. Additionally, the idea that the spa is a pleasant place should only be used in marketing materials for wellness tourists, as it is not important for healthcare/medical spa tourists. Given the scant role played by social influence in the decision to go to spas in both segments, efforts should not be expended to convince potential spa-goers’ friends and family members of the benefits of spas.

This research has several limitations that should be taken into account when considering the results. First, the data were collected in relation to a single spa. This decision was made to prevent the influence of uncontrollable variables. However, this means that the results only refer to that spa; it is not known whether they would be the same for other facilities with different characteristics. Second, the sample consisted of only potential Spanish tourists. It is thus likewise not known whether the results would be the same for tourists from other countries. Future research should include a broader comparative study between establishments and countries.

## Figures and Tables

**Table 1:** Technical details of the data collection and sample characterization.

Research Universe	Spanish Residents Who Do Not Live in Pantón
Data collection method	Quantitative survey, administered face-to-face or by phone
Sample	810 people
Data collection period	March and April 2019
Sample characteristics	
Gender	Men: 50% Women: 50%
Age	20 to 30 years old: 20% 31 to 40 years old: 20% 41 to 50 years old: 20% 51 to 60 years old: 20% 61 years old or more: 20%
Formal education	No formal education: 3.5% Primary school: 27.7% Secondary school: 32.2% Higher education: 36.5%

**Table 2:** Scale items used to measure the model’s variables.

Construct	Items	Source
Performance Expectancy (PE)	PE1. Using the thermal suite would be useful to me PE2. Using the thermal suite would increase my chances of achieving things that are important to me PE3. Using the thermal suite would help me achieve my goals faster PE4. Using the thermal suite would increase my quality of life	[19]
Effort Expectancy (EE)	EE1. It will be easy for me to learn how to use the thermal suite EE2. For me, how to use the thermal suite will be clear and comprehensible EE3. It will be easy for me to use the thermal suite EE4. It will be easy for me to be an expert in using the thermal suite	
Social Influence (SI)	SI1. The people who are important to me would think that I should use the thermal suite SI2. The people who influence me would think that I should use the thermal suite SI3. The people whose opinions I value would like me to use the thermal suite	
Pleasure (PL)	PL1. Unhappy–Happy PL2. Annoyed–Pleased	[79]
Arousal (AR)	AR1. Relaxed–Stimulated AR2. Calm–Excited	
Purchase Intention (PI)	PI1. If I went to the area, I would intend to use the thermal suite PI2. If I went to the area, I predict that I would use the thermal suite	[21]

**Table 3:** Standardized loading values (*t*-values) of the Cognitive-Affective-Normative (CAN) dimensions and purchase intention for wellness and healthcare/medical tourists.

Construct	Wellness	Healthcare/Medical
Performance Expectancy		
PE1	0.886 (34.15)	0.801 (16.79)
PE2	0.804 (63.64)	0.821 (20.47)
PE3	0.779 (29.53)	0.783 (23.67)
PE4	0.764 (24.51)	0.777 (19.26)
Effort Expectancy		
EE1	0.839 (11.61)	0.835 (7.29)
EE2	0.934 (14.27)	0.804 (12.51)
EE3	0.856 (12.24)	0.884 (15.62)
EE4	0.662 (6.643)	0.881 (10.81)
Pleasure		
PL1	0.925 (21.75)	0.896 (20.04)
PL2	0.803 (15.94)	0.897 (16.81)
Arousal		
AR1	u.i.	u.i.
Social Influence		
SI1	0.932 (45.45)	0.906 (29.99)
SI2	0.921 (41.71)	0.951 (53.76)
SI3	0.924 (49.92)	0.959 (41.76)
Purchase Intention		
PI1	0.909 (49.12)	0.908 (25.18)
PI2	0.923 (38.61)	0.937 (36.08)

Note. u.i. = unique item.

**Table 4:** Composite reliability, Cronbach’s alpha, average variance extracted (AVE) (convergent validity), and discriminant validity.

Construct	Composite Reliability > 0.7	Cronbach’s Alpha	AVE > 0.5	PE	EE	P	A	SI	PI
WELLNESS									
Performance Expectancy (PE)	0.89	0.88	0.66	**0.81**	0.19	0.32	0.25	0.73	0.67
Effort Expectancy (EE)	0.90	0.90	0.69	0.19	**0.83**	0.39	0.08	0.26	0.33
Pleasure (P)	0.85	0.86	0.75	0.32	0.39	**0.87**	0.04	0.32	0.45
Arousal (A)	1.00	1.00	1.00	0.25	−0.07	−0.03	**1.00**	0.19	0.13
Social Influence (SI)	0.95	0.95	0.86	0.73	0.26	0.32	0.19	**0.93**	0.53
Purchase Intention (PI)	0.91	0.91	0.84	0.68	0.33	0.45	0.13	0.53	**0.92**
HEALTHCARE/MEDICAL									
Performance Expectancy	0.87	0.87	0.63	**0.80**	0.47	0.51	0.09	0.77	0.71
Effort Expectancy	0.91	0.91	0.73	0.47	**0.85**	0.37	0.05	0.44	0.39
Pleasure	0.89	0.89	0.80	0.51	0.37	**0.90**	0.14	0.39	0.44
Arousal	1.00	1.00	1.00	0.09	−0.02	−0.14	**1.00**	0.02	0.02
Social Influence	0.96	0.96	0.88	0.77	0.44	0.39	0.02	**0.94**	0.56
Purchase Intention	0.92	0.92	0.85	0.71	0.39	0.44	−0.01	0.56	**0.92**

Note: Diagonal elements (in bold) are the square root of the AVE. Off-diagonal elements are the correlations among the constructs. The elements above the diagonal (in bold) are the HTMT values.

**Table 5:** Multigroup comparison.

Construct	Path Coefficients-Diff. (Wellness vs. Healthcare/Medical)	*p*-Value of the Henseler Test
Performance Expectancy ≥ (+) Purchase Intention	–0.04	0.60
Effort Expectancy ≥ (+) Purchase Intention	0.09	0.16
Pleasure ≥ (+) Purchase Intention	0.13	0.08
Arousal ≥ (+) Purchase Intention	0.05	0.21
Social Influence ≥ (+) Purchase Intention	–0.03	0.57
